# Relapsed/Refractory Multiple Myeloma: A Review of Available Therapies and Clinical Scenarios Encountered in Myeloma Relapse

**DOI:** 10.3390/curroncol30020179

**Published:** 2023-02-15

**Authors:** Parva Bhatt, Colin Kloock, Raymond Comenzo

**Affiliations:** Tufts University Medical Center, Boston, MA 02145, USA

**Keywords:** relapsed/refractory, multiple myeloma, immunomodulatory agents, proteasome inhibitors, monoclonal antibodies, autologous stem cell transplant, renal impairment, extramedullary disease, chemotherapy, Venetoclax

## Abstract

Multiple myeloma remains an incurable disease with the usual disease course requiring induction therapy, autologous stem cell transplantation for eligible patients, and long-term maintenance. Risk stratification tools and cytogenetic alterations help inform individualized therapeutic choices for patients in hopes of achieving long-term remissions with preserved quality of life. Unfortunately, relapses occur at different stages of the course of the disease owing to the biological heterogeneity of the disease. Addressing relapse can be complex and challenging as there are both therapy- and patient-related factors to consider. In this broad scoping review of available therapies in relapsed/refractory multiple myeloma (RRMM), we cover the pharmacologic mechanisms underlying active therapies such as immunomodulatory agents (IMiDs), proteasome inhibitors (PIs), monoclonal antibodies (mAbs), traditional chemotherapy, and Venetoclax. We then review the clinical data supporting the use of these therapies, organized based on drug resistance/refractoriness, and the role of autologous stem cell transplant (ASCT). Approaches to special situations during relapse such as renal impairment and extramedullary disease are also covered. Lastly, we look towards the future by briefly reviewing the clinical data supporting the use of chimeric antigen receptor (CAR-T) therapy, bispecific T cell engagers (BITE), and Cereblon E3 Ligase Modulators (CELMoDs).

## 1. Introduction

In the current era of multiple myeloma therapy, excellent treatment options exist for patients that result in survival of at least 5 years in about 55% of patients [1]. However, despite the use of triplet and quadruplet induction regimens, autologous stem cell transplant, and maintenance therapy, myeloma remains incurable, with relapses invariably occurring at different stages in the course of the disease. When relapse is encountered, management can be complex and challenging owing to the biological heterogeneity of the disease. Over the past two decades, a focus of translational and clinical research has been on overcoming drug resistance. Novel therapeutics are making it from bench to bedside at a rapid rate, resulting in better disease control and longer lives for our patients.

Relapsed and/or refractory disease as defined by the International Myeloma Working Group (IMWG) is useful for designing clinical trials [2]. “Refractory myeloma” is disease that is non-responsive to therapy or progresses within 60 days of the last line of therapy [2]. “Relapsed myeloma” is previously treated myeloma that has progressed after prior therapy and requires new therapy [2]. “Relapsed and refractory myeloma” is disease nonresponsive to the chosen line of therapy in patients who had achieved a minimal response or better at some point previously in their disease [2]. Lastly, “primary refractory myeloma” is disease that is nonresponsive to treatment in patients who never achieve a minimal response or better [2]. Similarly, we use the terms complete response (CR), very good partial response (VGPR), partial response (PR), minimal response (MR), stable disease (SD), and progressive disease (PD) as defined by the IMWG [2]. A summary of the IMWG response criteria is provided in Table 1.

**Figure 1:**
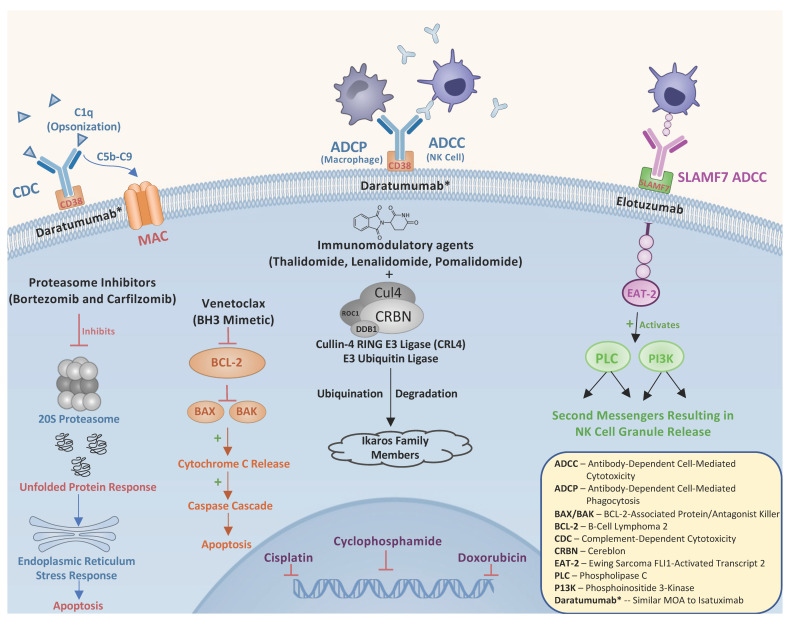
Summary of pharmacologic mechanisms of action of active therapies used in the treatment of RRMM.

In this review of RRMM we discuss the pharmacologic mechanisms underlying the commonly used agents in the relapse setting which include immunomodulatory agents (IMiDS), proteasome inhibitors (PIs), monoclonal antibodies (mAbs), traditional chemotherapy, and the BCL2 inhibitor Venetoclax (Figure 1). We also take a deep dive into the clinical data supporting treatment decisions when there is relapse/refractoriness to back-bone therapies such as Lenalidomide (LEN), Bortezomib (BOR), and Daratumumab (DARA). We also address unique circumstances to consider during relapse, including relapse with renal impairment, extramedullary relapse, and the role of a second autologous stem cell transplant (ASCT). Lastly, we briefly review the future direction RRMM therapy is headed, with a focus on cellular therapies such as chimeric antigen receptor T (CAR-T) cell therapy, bispecific T-cell engager (BITE) therapy, and Cereblon E3 Ligase Modulators (CELMoDs).

## 2. Overview of Available Therapies in RRMM

### 2.1. Immunomodulatory Drugs (IMiDs)

The introduction of immuno-modulatory drugs began with Thalidomide in the late 1990s for relapsed refractory multiple myeloma (RRMM) and revolutionized the treatment options offered to patients. A phase II study enrolling 84 RRMM patients led to the eventual approval of Thalidomide with Dexamethasone in 2006 by the FDA to treat both newly diagnosed multiple myeloma (MM) and RRMM [4,5]. The early success of Thalidomide prompted an investigation into additional IMiDs, the most notable of which are Lenalidomide and Pomalidomide.

The IMiDs share a common mechanism of action with newer generations showing added anti-myeloma activity and differing in pharmacologic properties such as half-life, liver metabolism, and the need for renal dosing [6,7]. As a class, IMiDs have a wide range of biological activities such as inhibition of NF-kB and interferon regulatory factor 4 (IRF4), increased expression of pro-apoptotic factors such as Caspase-8, and downregulation of angiogenesis via VEGF and IL-6 production leading to a disruption of the myeloma cell-marrow microenvironment interaction [8,9,10,11]. Disruption of the cytokines TNF-α, IFN-γ, IL-1b, IL-2, and IL-12 has been shown over the years as an additional mechanism of anti-myeloma activity [12,13]. More recently, stabilization of cereblon (CRBN), a component of the cullin-4 RING E3 ligase (CRL4) complex with E3 ubiquitin ligase activity, has been identified as the direct target of IMiDs promoting degradation of Ikaros (IKZF1) and Aiolos (IZKF3) transcription factors [14,15]. Aiolos, an Ikaros family member, is responsible for the generation of high-affinity plasma cells in the bone marrow and the degradation of these Ikaros family members downregulates IRF4 and upregulates IL-2 production as mentioned previously [16,17,18]. Zhu et al. identified over 46 CRBN binding proteins that were decreased during Lenalidomide treatment and found that IZKF1 levels correlate with IMiD responsiveness and overall survival [19]. Lenalidomide forms a molecular bridge between CRBN and Casein kinase 1α (CK1α) thereby promoting ubiquitination and degradation of CK1α [20]. CK1α promotes the survival and proliferation of MM as a pro-growth kinase and CK1α loss of function resulted in the downregulation of anti-apoptotic cascades yielding an anti-myeloma effect [21]. The downstream effects of IMiDs result in multiple myeloma cytotoxicity and are relatively well-tolerated.

In addition, immune dysregulation is a hallmark of MM by way of abnormal Th1/Th2 ratios, aberrant T cell function achieved via TGF-β secretion by MM cells, and immune suppression via disruption of Treg/Th17 balance [22]. IMiDs induce T cell proliferation and co-stimulation through INF-γ, IL-10, and IL-2 production. Dendritic cell (DC) activation through IMiD-enhanced DC-antigen presentation increases activation of CD4^+^/CD8^+^ T cells which promotes immune surveillance and an anti-myeloma profile [12]. Myeloma-induced exhaustion and senescence of T cells are seen in the bone marrow milieu. Lenalidomide maintenance has been shown to reduce programmed cell death protein 1 (PD-1) expression on CD8^+^ T cells which may be an additional mechanism by which IMiDs may reverse such senescence and exhaustion [23]. Lenalidomide’s ability to increase IFN-γ promotes a phenotypic shift to a Th1 profile that results in amelioration of the defective anti-tumor Th1 population seen in MM [24]. Myeloid-derived suppressor cells, Tregs, central memory CD8^+^ T cells, and effector memory CD8^+^ T cells were all increased following Lenalidomide treatment—suggesting immunomodulation on many different lymphoid compartments [25].

### 2.2. Proteasome Inhibitors

Phase 1 data regarding first-generation proteasome inhibitors (PIs) was first published in 2002 and paved the way for the eventual approval of Bortezomib in 2003 for RRMM [26]. Data from the SUMMIT and APEX trials helped solidify Bortezomib as an integral part of MM therapies beyond just RRMM [26,27,28]. Additional approval for second and third-generation PIs, Carfilzomib and Ixazomib, would come over the next few years with Carfilzomib’s approval for RRMM in 2012 and Ixazomib in 2015. Ixazomib differs from its predecessors in that it is given orally, an attractive option for elderly/frail patients or those looking to limit their time spent in the medical setting. The selection between Carfilzomib compared to Bortezomib takes into consideration the side effect profile with Carfilzomib having a measure of cardiotoxicity whereas Bortezomib can cause painful but reversible peripheral neuropathy in 10% to 25% of patients even when dosed subcutaneously on a weekly basis.

The pharmacology of PIs is related to their ability to inhibit NF-kB signaling through both canonical and non-canonical pathways via proteasome inhibition of IkB and p100/105 proteins, respectively [29]. Downstream anti-myeloma, pro-apoptotic effects of attenuated NF-kB activity are related to NF-kB’s ability to regulate caspase inhibitors, Bcl-2 family members, cytochrome c extrusion from mitochondria, and cytokine production [30,31]. The critical anti-myeloma activity of PIs, however, stems from the accumulation of misfolded proteins that would normally be degraded by the 20S proteasome. This accumulation yields an apoptotic response via endoplasmic reticulum stress termed the terminal unfolded protein response [32,33].

### 2.3. Monoclonal Antibodies

The accelerated approval of Daratumumab, an anti-CD38 IgG1 monoclonal antibody, in 2015 for the treatment of MM marked the beginning of an era utilizing monoclonal antibodies to treat heavily treated MM patients [34]. This approval was limited to the addition of two doublet therapies: Lenalidomide with Dexamethasone and Bortezomib with Dexamethasone. Within the anti-CD38 family, Isatuximab was first granted approval in 2020 for use in RRMM with Pomalidomide and Dexamethasone [35]. The function and structural elucidation of Signaling Lymphocytic Activation Molecule Family Member 7 (SLAMF7) helped pave the way for a new monoclonal antibody target and was approved by the FDA in 2015 for use with Lenalidomide and Dexamethasone in those who have had previous treatment for MM.

There are several mechanisms by which Daratumumab functions as an anti-myeloma drug: antibody-dependent cell-mediated cytotoxicity (ADCC), complement-dependent cytotoxicity (CDC), and antibody-dependent cellular phagocytosis (ADCP) [36,37,38,39]. Oncell-driven of NK cell driven ADCC occurs through the granule-exocytosis pathway that includes membrane-disrupting perforins and granzymes resulting in the activation of caspase with subsequent apoptosis [40]. Complement-dependent cytotoxicity is hallmarked by opsonization with C1q, triggering the classical complement pathway which ultimately leads to the formation of the membrane attack complex (MAC) via C5b-C9 deposition into the cell membrane [41]. Daratumumab-dependent macrophage-mediated phagocytosis has been reported and is a function of being opsonized by Daratumumab on the CD38 receptor [42]. In addition to the anti-CD38 effects of Daratumumab, Isatuximab has an interesting pro-apoptotic effect by means of increased lysosomal membrane permeability, lysosomal enlargement, generation of reactive oxygen species, and subsequent extrusion of cathepsin B resulting in lysosome-dependent cell death [43,44]. 

Hsi et al. found that SLAMF7 was expressed in a variety of immune cells including malignant hematopoietic cells, CD8 T cells, B cells, monocytes, dendritic cells, and most importantly, NK cells [45]. Non-lymphoid tissue was spared and does not express SLAMF7. SLAMF7 is continued to be expressed in patients with MM who have undergone treatment, but also in those who have asymptomatic MM such as smoldering MM and MGUS [45,46]. Elotuzumab is a humanized, IgG1 anti-SLAMF7 monoclonal antibody that functions to activate SLAMF7^+^ NK cells directly towards SLAMF7^+^ MM cells and induces dose-dependent ADCC similar to Daratumumab without cytotoxicity to autologous SLAMF7^+^ NK cells [47,48]. Not only does Elotuzumab promote NK cell to MM cell conjugation, but it appears to augment the granzyme release potential of NK cells to further promote ADCC against MM cells [48]. Downstream intracellular signaling of SLAMF7 occurs via phosphorylation of SLAMF7 by Ewing’s sarcoma’s/FLI1-activated transcript 2 (EAT-2) with second messengers phospholipase C (PLC) and phosphoinositide 3-kinase (PI3K) accomplishing intracellular amplification of downstream targets resulting in granule release [47,49,50,51]. Although Elotuzumab does not have single-agent activity in RRMM, it is active in combination with Lenalidomide based on phase III trial data [52].

### 2.4. Chemotherapy

Traditional chemotherapy utilized in several malignancies is also used in MM in combination with therapies such as DT-PACE and CyBorD. The combination of Dexamethasone, Thalidomide, and the continuous infusion of Cisplatin, Doxorubicin, Cyclophosphamide, and Etoposide is coined DT-PACE. An additional and highly utilized regimen of Cyclophosphamide, Bortezomib, and Dexamethasone (CyBorD) is available for use and was recently trialed with Daratumumab with a durable response [53]. The alkylating agent Melphalan continues to be the conditioning regimen of choice for autologous hematopoietic stem cell transplants (HSCTs) although recent trials have looked at the addition of Bendamustine to Melphalan conditioning prior to auto-HSCT [54].

The use of chemotherapy in combination therapy is based on synergy with each other. Cisplatin achieves cytotoxicity through the covalent binding of platinum to guanine and adenine resulting in intra and inter-strand crosslinking that promotes strand breaks within the DNA which results in cell apoptosis [55]. Hepatic metabolism of Cyclophosphamide yields the active alkylating agent phosphoramide mustard which forms permanent, irreversible cross-linkages between adjacent DNA strands that leads to cell apoptosis. Cyclophosphamide also has immunosuppressive effects and decreases INF-γ with increases in IL-4 and IL-10 [56]. Doxorubicin, an anthracycline chemotherapeutic with a well-documented delayed irreversible cardiomyopathy side effect profile, inhibits topoisomerase II and intercalates within DNA base pairs causing DNA damage and subsequent cell apoptosis [57]. Etoposide, functioning primarily in the late S and G2 phases, inhibits topoisomerase II as well and triggers apoptosis [58].

### 2.5. Venetoclax

Historically having been approved for chronic lymphocytic leukemia and acute myeloid leukemia, Venetoclax has found a new use in those MM patients harboring a t(11:14) mutation [59]. As an oral BH3 mimetic and BCL-2 inhibitor, Venetoclax inhibits the anti-apoptotic protein BCL-2 which promotes mitochondrial permeability with subsequent caspase activation via pro-apoptotic BAX/BAK signaling pathways [60,61,62].

### 2.6. Selinexor

Selinexor, is a first-in-class oral selective inhibitor of nuclear export (SINE), currently, FDA approved in combination with Bortezomib and Dexamethasone for patients having received one prior therapy [63]. Selinexor reversibly inhibits the nuclear export function of Exportin-1 (XPO1), a protein that is responsible for shuttling over 200 macromolecules out of the nucleus [64]. Selinexor binds the leucine-rich nuclear export signal (NES) found in the karyopherin XPO1. Also known as Chromosomal Maintenance 1 (CRM1), XPO1 inhibition blocks the exporting of oncogene mRNAs such as c-myc resulting in a reduction of oncoproteins [65]. Additional anti-myeloma effects of Selinexor occur through reactivation of tumor suppressor proteins (TSPs) such as p53, sensitization of the glucocorticoid receptor to Dexamethasone, inhibition of the mTOR pathway, and retention of inhibitor of NF-κB (lκB) [66,67,68]. Retention of lκB inhibits NF-κB signaling—a known pathway involved in myeloma cell survival. Kashyap et al. documented the synergy of SINE compounds with proteasome inhibitors through inhibition of the phosphorylation of IκB and NF-κB subunits thereby protecting IkB from proteasome degradation. This results in NF-κB suppression and a subsequent increase in the cytotoxicity of myeloma cells seen both in vitro and in vivo [69]. There is relative sparing of non-malignant cells by tumor suppressor protein (TSP)-induced apoptosis as TSPs induce apoptosis in cells with significant DNA damage. This first-in-class SINE compound offers several new mechanisms against MM and may elucidate additional means of synergy among myeloma treatments.

### 2.7. CAR-T/BITE Therapy

The molecular mechanisms by which CAR-T and BITE therapies are effective in treating myeloma are largely based on the interaction of the malignant plasma cell with autologous T-cells. Multiple cell surface proteins expressed on plasma cells are targets for drug development with the most notable being B-cell maturation antigen (BCMA), a transmembrane, non-tyrosine kinase, glycoprotein. BCMA is an ideal target to inhibit as it is not only preferentially expressed on plasma cells with minimal expression in stem cells or non-hematopoietic tissue but is also needed for the survival of bone marrow plasma cells [70]. Furthermore, overexpression and activation of BCMA are associated with the progression of myeloma in preclinical models and humans via canonical and non-canonical NF-kB pathways in charge of cell survival, growth, and metastasis [70].

BCMA CAR constructs contain an extra-cellular component derived from an immunoglobulin heavy and light chain variable domains that link to form a single chain variable fragment (scFv) capable of recognizing BCMA [71]. A hinge or spacer domain is then linked to an intracellular CD3-zeta signaling chain of the T-cell receptor which provides the first signal for activation of the T cell [71]. Additionally, to promote CAR-T cell survival and proliferation, additional costimulatory domains are incorporated into the construct, which in the case of idecabtagene vicleucel (ide-cel) and ciltacabtagene autoleucel (cilta-cel), the costimulatory domain is 4-1bb [71]. Subsequent tumor killing is mediated by activated CAR-T cell mediated tumor killing by (1) granzyme and perforin-mediated cytotoxicity (2) cytokine release to sensitize tumor stroma for target cell death, and (3) Fas/FasL mediated activation of caspase-mediated cellular apoptosis [72].

BITE therapies are recombinant proteins that contain two separate linked single-chain variable fragments (scFv) which can simultaneously bind to a tumor cell and an immune effector cell to generate an immune synapse between the two [73]. In the case of BCMA-directed BITEs the scFv recognizes BCMA on the plasma cell and CD3 on the T-cell [73]. Downstream effects of T cell activation are similar to what is seen with CAR-T cell therapy in that tumor killing is mediated by granzyme/perforin, cytokine release, and caspase-mediated apoptosis. The added benefit of BITEs involves the upregulation of multiple T-cell compartments, both CD4^+^ and CD8^+^, leading not only to myeloma cell lysis but also differentiation of naïve T cells into memory T cells as well [73].

## 3. Clinical Scenarios

### 3.1. Relapse Due to Lenalidomide Resistance/Refractoriness

Lenalidomide is an established backbone of treatment for myeloma in both the front-line and maintenance settings. The development of resistance to lenalidomide is thought to occur due to the consequence of mutations in key signaling pathways in plasma cell clones that emerge after initial therapy [74]. Multiple clinical trials have evaluated strategies to overcome resistance involving continued immunomodulation with Pomalidomide, class switching to other active agents, or the use of monoclonal agents. Table 2 summarizes the outcomes of ORR, PFS, and OS in the LEN-resistant/refractory subgroups from key clinical trials discussed in this section.

#### 3.1.1. Immunomodulation with Pomalidomide

In both doublet and triplet combinations, Pomalidomide has shown efficacy in the treatment of RRMM; however, clinical trials have varied in terms of the prior lines of therapy patients received prior to the introduction of POM. As a doublet, the Phase III MM-003 (NIMBUS) compared POM-loDEX vs. HiDEX and MM-010 (STRATUS) evaluated the effectiveness of POM-loDEX in a Phase 3b study [75,76]. In the MM-003 study, the majority of patients were refractory to lenalidomide (~93%), with close to 75% being refractory to both LEN and BOR [75]. In the LEN refractory patients, superior ORR, PFS, and OS were all seen in the POM-loDEX as compared to HiDEX [75]. Similarly, in the dual LEN/BOR refractory patients better PFS was seen but OS did not reach statistical significance [75]. In the MM-010 study the majority of patients were refractory to lenalidomide (95%) or dual refractory to LEN/BOR (80%). At a median follow-up of 16.8 months, ORR, PFS, and OS were all of a similar magnitude to that seen in MM-003 with additional evidence supporting the safety and tolerability of the combination of POM-loDEX [76].

As a triplet, the combination of POM-BOR-Dex was compared to BOR-dex in the phase III OPTIMISMM study in which the study population was enriched for patients considered LEN refractory, approximately 70% in the ITT population with 100% of patients having prior LEN exposure [77]. At median follow-up, PFS was improved in the POM-BOR-Dex group as a whole (11.2 vs. 7.1 months, *p* < 0.0001) as well in the LEN refractory patients (9.5 vs. 5.6 months, *p* = 0.0008) [77]. Overall survival data were not mature at the time of planned interim analysis [77]. In a more recent sub-group analysis by the study investigators, the PFS benefits of POM-BOR-dex were confirmed in patients at both first relapse and with high-risk cytogenetic abnormalities [78].

When considering the introduction of POM either as a doublet or triplet, the aforementioned clinical trials vary with respect to prior lines of therapy. In the STRATUS and NIMBUS trials, patients had received a median of five prior lines of therapy suggesting a role for POM in heavily pre-treated populations [75,76]. In contrast, in the more recently published OPTIMISMM, close to 80% of patients had received 1–2 prior lines of therapy and 57% of patients had undergone autologous stem cell transplant [77]. These differences highlight the role of additional immunomodulation with POM as a strategy to overcome LEN refractoriness in both early or late relapse and in heavily pre-treated patients.

#### 3.1.2. Use of Monoclonal Antibody therapy

Employing monoclonal antibody therapy with agents such as Daratumumab (DARA), Isatuximab (ISA) or Elotuzumab (ELO) either as a class switch strategy or in combination with POM are additional approaches that have been employed in the LEN refractory setting. There have been several clinical trials evaluating triplet combinations of a monoclonal agent plus proteasome inhibition and steroids. Where these trials differ is in the proportion of patients in each study considered LEN refractory. Given the activity of these monoclonal agents in the relapse setting, treatment decisions are often made based on pre-existing co-morbidities, prior drug exposure, patient tolerance, and insurance considerations

With respect to DARA, the CASTOR and CANDOR trials assessed the combination of DARA with either bortezomib or carfilzomib, respectively. In the Phase 3 CASTOR trial, patients were randomized to either DARA-BOR-dex versus BOR-dex with a crossover design in which patients in the control arm could receive DARA on progression [79]. Patients had received a median of two prior lines of therapy with 24% of patients in the experimental arm considered LEN refractory [79]. At final analysis with a median of 72 months of follow-up, significant mOS benefit was seen in the DARA-BOR-dex arm in the intention to treat population (ITT) (49.6 vs. 38.5 months, HR 0.75, *p* = 0.0075) [80]. This benefit was most pronounced in patients having received oneprior line of therapy [80]. In the pre-specified LEN refractory sub-group, there was a statistically significant improvement in mPFS and ORR; however, at final analysis, OS in this subgroup was not found to be statistically significant [80,81]. This difference could be attributable to the crossover design of the trial with approximately 35% of patients having received DARA at progression in the control arm. Phase 3 CANDOR employed proteasome inhibition with carfilzomib (K) in combination with DARA and dex (DARA-Kd) versus Carfilzomib-dex (Kd) with a similar study population as compared to CASTOR [82]. In this study, patients had received a median of two prior lines of therapy with approximately 32–36% of patients considered LEN refractory [82]. In the most recent analysis of this study, at a median follow-up of 27.8 months, the median PFS in the Dara-Kd versus Kd arm was 28.6 months versus 15.2 months for the ITT population (HR 0.59, *p* < 0.0001) [83]. Subgroup analysis of the LEN refractory patients showed a statistically significant PFS difference between groups [83]. Overall survival data from CANDOR is not yet mature.

With respect to ISA, the phase 3 IKEMA study compared the combination of ISA with Carfilzomib-dex (ISA-Kd) versus Kd as a control [84]. In this study, patients had received a median of 2 prior lines of therapy with 32% of patients being refractory to LEN [84]. At a median follow-up of 20 months, in the ITT population the median PFS was not reached in the ISA-Kd group versus 19.15 months in the control arm (HR 0.53, *p* = 0.0007) [84]. Similarly, in the pre-specified subgroup analyses of LEN refractory patients, mPFS was not reached compared to the control group, however likely due to the small sample size, this finding did not reach statistical significance [84]. Similar to CANDOR, the overall survival data is not yet mature.

In terms of ELO, an initial phase 2 open label trial comparing ELO-BOR-dex to BOR-dex was completed with a primary endpoint of PFS. Based on a pre-specified significance level of *p* ≤ 0.3 the study did meet its primary endpoint, (mPFS 9.7 vs. 6.9 months, *p* = 0.09); however, these results are signal generating and require larger randomized trials to validate [85]. Additionally, whether these findings apply to LEN refractory patients is yet to be determined. 

In summary, the combination of Anti-CD38 monoclonal antibodies with proteasome inhibition and steroids shows efficacy in the RRMM setting in terms of PFS and ORR; however, an OS benefit is yet to be seen. Furthermore, it is important to note that subgroups of LEN refractory patients in these studies were relatively small and PFS benefit did not consistently reach statistical significance suggesting that these combinations may not be preferable for this group of patients. 

The strongest evidence pointing towards clinical benefit in the use of monoclonal antibody therapy is in combination with POM-dex (Pd), specifically the Phase 3 APOLLO (Dara-Pd), Phase 3 ICARIA (Isa-Pd), and Phase 2 ELOQUENT-3 (Elo-Pd) studies. In all of these trials, patients had received a median of 2–3 lines of prior therapy and were compared to a control arm of POM-dex [86,87,88]. In each trial, the percentage of patients having received prior Lenalidomide exceeded at least 80% and nearly all patients were considered LEN refractory [86,87,88]. In APOLLO and ICARIA, primary endpoint of PFS was met, 12.4 months (*p* = 0.0018) in APOLLO and 11.6 months (*p* = 0.001) in ICARIA, with control arms in both studies performing similarly with PFS of 6.9 and 6.5 months on POM-dex alone [86,89]. In subgroup analyses of both trials, mPFS and ORR were better for LEN-refractory, BOR-refractory, and double-refractory patients consistent with benefits seen in the overall study population [86,90].

In ICARIA specifically, the most recent survival data from the second planned interim analysis, published in March 2022, showed an OS difference of 6.9 months in the Isa-Pd versus the POM-dex control (23.6 mos vs. 17.7 mos, HR 0.76, *p* = 0.028) at a median follow up of 35.3 months [91]. Final OS analysis for this study is still pending but given the safety profile of Isa-Pd in the clinical trial we expect continued survival benefits as data mature. While overall survival data has yet to mature for patients receiving Dara-Pd in APOLLO, a recent publication of patient-reported outcomes (PROs) showed substantial improvement in pain, functional status, and disease-related symptoms compared to POM-dex [92]. How these findings of efficacy and safety persist over a longer duration of follow-up and in the real-world setting continues to be assessed. Nevertheless, the combination of Anti-CD38 monoclonal antibody therapy with DARA or ISA in combination with POM is a powerful strategy in the setting of myeloma relapse with LEN resistance.

Interestingly, in ELOQUENT-3, at 45 months of follow-up, there was sustained, statistically significant separation of the curves in terms of OS for Elo-Pd versus Pd (29.8 mos vs. 17.4 mos, HR 0.59, *p* = 0.0217) [88]. For the LEN and PI dual-refractory subgroup, while there was not a statistically significant benefit, there was a trend towards improved median OS [88]. Studies with larger sample sizes evaluating SLAMF7 inhibition with ELO are needed to determine whether these overall survival findings persist in LEN refractory patients.

#### 3.1.3. Switching to a Different Class of Medication

Phase 3 clinical trials involving drugs with novel mechanisms of action (excluding IMIDs, PIs, and monoclonal Ab) demonstrating benefit in LEN refractory RRMM remain areas of intense ongoing research. Further discussion regarding the role of cellular therapies targeting BCMA such as CAR-T and BITE therapies will be discussed in a later section of this review. The nuclear transport inhibitor, Selinexor (SEL), and the BCL-2 inhibitor Venetoclax (VEN), are two drugs with a role, particularly in the setting of early relapse.

Regarding Selinexor, in the Phase 3 BOSTON trial patients were randomized to a combination of once-weekly SEL-BOR-dex (SVd) versus BOR-dex (Vd) with patients having received a median of one prior line of therapy [93]. In this trial approximately 39% of patients previously received LEN; however, they were not specified to be LEN refractory [93]. In the ITT population, at a median follow-up of 13.2 months, the primary endpoint of mPFS was met (13.93 vs. 9.46 months, HR 0.70, *p* = 0.0075). In the subgroup of patients with previous LEN exposure, the hazard ratio would suggest improved PFS but PFS duration or statistical significance was not reported [93]. The findings from the BOSTON and the lead-up phase 2 study STORM led to the FDA approval of this medication for RRMM having received at least one prior line of therapy [93,94]. Whether, SEL has a role in 2nd or 3rd line of therapy in the face of regimens such as DARA-IMID or ISA-IMID is up for debate. There is hesitancy to reach for SEL as there are questions as to whether the BOSTON trial selected an appropriate control arm in BOR-dex as this doublet combination is virtually never pursued in the relapse setting. Additionally, a significant proportion of patients experienced serious adverse events (52%) or needed dose modifications (89%) raising questions about the tolerability of SEL and whether once weekly is an appropriate dosing schedule [93]. Additional data regarding the appropriate dosing and combinations of SEL are needed to establish its role for the approved FDA indication.

Venetoclax, while not currently FDA-approved for RRMM, may have a role, specifically in patients with t(11;14) or high BCL-2 expression. The phase 3 BELLINI study compared VEN-BOR-dex (Ven-Vd) versus Vd in patients having received a median of one prior line of therapy [95]. Twenty percent of patients were considered refractory to LEN in this study [95]. At median follow-up of 18.7 months, median PFS was significantly longer in the VEN-Vd versus control (22.4 mos vs. 11.5 mos, HR 0.63, *p* = 0.010) for the ITT population [95]. However, in the LEN refractory group statistical significance was not seen [95]. In terms of overall survival, VEN-Vd performed worse than Vd and per the study investigators’ potential explanations included increasing toxicity and risk of infection, limited use of prophylactic antibiotics, and potential selection of aggressive malignant plasma cell clones in patients without t(11;14) and low BCL-2 expression [95]. When evaluating the role of Venetoclax in LEN refractory patients, the deciding factor becomes the presence of t(11;14), as this subgroup of patients did demonstrate statistically significant improvement in PFS (HR 0.11, *p* = 0.004) and OS (HR 0.24, *p* < 0.0001), and ORR [95]. 

### 3.2. Relapse Due to Bortezomib Resistance/Refractoriness

Often when patients develop Bortezomib resistance/refractoriness, lenalidomide resistance/refractoriness is also encountered due to the nature of triplet and quadruplet therapies in the frontline setting incorporating both agents. The same strategies used to approach LEN resistance can also be used to approach BOR resistance; however, clinical trials that contain significant proportions of patients that are specifically BOR-resistant or dual PI-IMID resistant are lacking. These strategies involve the introduction of a second-generation proteasome inhibitor such as Carfilzomib (CAR) or Ixazomib (IXA), class-switching to a different class of drug with a novel mechanism, or the introduction of monoclonal antibody therapy. The presence or absence of specific cytogenetic abnormalities, previous therapy, and weighing risks/benefits in shared decision-making, guide treatment selection in this setting. Table 3 summarizes the outcomes of ORR, PFS, and OS in subgroups of patients with previous BOR exposure from key clinical trials discussed in this section.

The Phase 3 ASPIRE and ENDEAVOR trials established the role of Carfilzomib in the RRMM setting [96,97]. In ASPIRE, patients were randomized to a combination of CAR-LEN-dex (KRd) versus LEN-dex (Rd), with a median follow-up of 67.1 months at final analysis [96,98]. For the ITT population, this study met all three major prespecified endpoints of median OS (48.3 mos vs. 40.4 mos, HR 0.79, *p* = 0.0045), median PFS (26.1 mos vs. 16.6 mos, HR 0.66, *p* < 0.001), and ORR [98]. Furthermore, specifically in patients with prior BOR exposure who had received one prior line of therapy (67%), median OS was improved by 12 months [98]. 

In the ENDEAVOR study, CAR was compared head-to-head against BOR, both arms in combination with Dex [97]. Patients in this study had received a median of two prior lines of therapy, with 54% of patients having received prior Bortezomib as part of front-line therapy [97]. At median follow-up of approximately 12 months, CAR-dex was superior to BOR-dex in terms of mPFS (18.7 mos vs. 9.4 mos, HR 0.523, *p* < 0.0001) [97]. Updated OS data at median follow-up of 44 months showed clinically meaningful improvement in survival for CAR-dex over BOR-dex (47.8 mos vs. 38.8 mos, HR 0.76, *p* = 0.0017) [99]. These findings were consistent across a variety of subgroups including age, cytogenetic risk, and prior bortezomib exposure, among others [99].

The oral proteasome inhibitor Ixazomib (IXA) was studied in the TOURMALINE MM1 trial in which the combination of IXA-LEN-dex (IRd) was compared to LEN-dex (Rd) with primary endpoint of PFS [100]. Patients had received up to two prior lines of therapy with 69% having received prior BOR therapy [100]. At primary analysis at a median follow-up of 14.8 months, mPFS favored the IRd group (20.6 vs. 14.7 mos, HR 0.74, *p* = 0.01) [100]. Overall survival data were not mature at the time; however, updated survival data published in 2021, with median follow-up of 85 months, did not show an OS difference (53.6 vs. 51.6 mos, HR 0.939, *p* = 0.495) [101]. Similar findings were seen in virtually all subgroups including patients with prior PI or IMID exposure [101]. Since TOURMALINE MM1 reported the longest median OS data of most clinical trials in RRMM, study investigators attributed this lack of difference in OS to subsequent post-protocol therapies, particularly DARA, providing extended survival in these patients [101].

Both the ASPIRE and ENDEAVOR trials inform decision-making as to whether Carfilzomib would be an appropriate choice in the RRMM setting, acknowledging its activity in patients having progressed on BOR, and side effect profile. It is clear from the ENDEAVOR data that switching to Carfilzomib is superior to re-treatment with Bortezomib. Ixazomib in combination with LEN-DEX provides an interesting, all-oral option for patients; however, survival data does not suggest sustained benefit long term. Additionally, to date, there have not been prospective randomized trials comparing Ixazomib to Carfilzomib or Bortezomib.

Clinical trials previously mentioned in the context of LEN resistance, also apply to the BOR-resistant or dual PI-IMID resistant setting from the standpoint of incorporation of monoclonal antibody therapy or class-switching. Significant proportions of patients were considered BOR or PI refractory in the APOLLO, ICARIA, and ELOQUENT-3 studies. In APOLLO there was a trend towards improved PFS in both the BOR and dual refractory subgroups [86]. Similar findings were seen in ICARIA favoring Isa-Pd and in ELOQUENT-3 for the dual-refractory subgroups [84,88]. 

Logically, given the efficacy of KRd, combinations of Carfilzomib-POM-dex or Carfilzomib-Cyclophosphamide-dex (KCyd) have also shown benefit in settings of BOR or dual BOR-LEN resistance, in Phase 2 trials. The final report from the Phase 2 EMN011/HOVON114 Trial, in which patients refractory to BOR and LEN received KPd, demonstrated an ORR of 92%, mPFS of 26 months, and mOS of 67 months [102]. Similarly, Mateos et al. (2020) demonstrated the combination of KCyd was effective (mPFS 20.7 months, ORR 78%) in patients having previously received a PI; however, in this Phase 2 study patients classified as refractory to BOR were excluded [103]. Class switching to Cy-POM-dex (CyPd) or Cy-LEN-dex (CyRd) has also been shown to be effective in early-phase studies. Garderet et al., showed that CyPd was effective at first relapse after induction RVD as bridge to Auto-SCT with up to 94% of patients achieving a PR or better [104]. Nijhof et al., showed in a Phase 1/2 study of heavily pretreated, multi-drug refractory patients that the combination of CyRd achieved an ORR of 67%, mPFS 12.1, and mOS of 29 months, respectively [105]. Findings from these studies highlight the variety of options currently available to patients refractory to BOR, LEN, or both.

### 3.3. Relapse Due to Daratumumab Resistance/Refractoriness

Daratumumab is currently approved for the treatment of multiple myeloma as both parts of initial induction and for relapsed disease. NCCN Consensus recommendations for induction regimens containing DARA for transplant-eligible patients include Dara-VTd, Dara-RVd, Dara-KRd, and DaraCyBorD. For transplant-ineligible patients, options include DaraRd, Dara-VMP, or DaraCyBorD. Given the more frequent use of DARA, resistance has inevitably emerged, and given the use of both IMID- and PI-containing regimens, it is becoming increasingly difficult to delineate which agent an individual patient with myeloma may be less responsive to. Furthermore, most Phase 3 clinical trials lack substantial subgroups of patients identified as DARA refractory making decision-making difficult. Reasonable strategies include re-treatment with DARA in cases of late relapse, or introduction of second-generation IMIDs or PIs depending on prior treatment exposure. Particularly in cases of early relapse after 1–2 prior lines of therapy, consideration of enrollment in clinical trials with novel cellular therapies such as CAR-T or BITE therapy should be considered. Table 4 summarizes the outcomes of ORR, PFS, and OS in subgroups of patients with DARA resistance/refractoriness from key clinical trials discussed in this section.

In situations where LEN or BOR are not implemented in first-line therapy, then logical choices include second- or third-line regimens that contain those agents in combination with DARA. Retreatment with DARA in combination with IMiDs specifically has been shown to overcome refractoriness to either agent in the frontline setting. For instance, Nooka et. al. performed a study in a series of 34 patients, the majority of whom were LEN and/or BOR refractory, separated into two cohorts-DARA and POM naïve or DARA-POM refractory [106]. In both cohorts they were able to demonstrate that patients were able to achieve a clinical response of PR or better; specifically, one-third of the DARA-POM refractory patients responded to DARA re-treatment [106].

The antibody drug conjugate Belantamab Mafodotin (BEL) received accelerated FDA approval for RRMM based on results from the DREAMM-2 study published in 2020 [107]. In DREAMM-2, patients were randomized to two separate dose intensities for single-agent BEL, heavily pre-treated with a median of 6–7 prior lines of therapy, and nearly all patients refractory to LEN, BOR, and DARA [107]. The median duration of response was not reached and 31–34% of patients achieving a partial response or better [107]. The subsequent phase 3 study, DREAMM-3 evaluated BEL versus POM-dex with primary endpoint of PFS and secondary endpoint of OS (NCT04162210). Unfortunately, the primary endpoint was not met [108]. Per initial analysis at a median follow-up of approximately 1 year, PFS for BEL versus POM-dex was 11.2 and 7 months, respectively, HR 1.03, with no significant differences seen in terms of ORR or OS [108]. Consequently, BEL has been withdrawn from the US market while additional clinical trials are ongoing to assess the best combinations of therapy.

More recently, Teclistamab (TEC), a bispecific T-cell engager, targeting BCMA on the plasma cell and CD3 on T cells, was studied in the Phase 1–2 MajesTEC-1 trial which led to its FDA approval for RRMM in patients experiencing progression after 3 prior lines of therapy including an Anti-CD38, IMID, and PI [109,110]. Patients in this study demonstrated deep and durable responses with approximately 39% of patients achieving a CR or better and 26.7% achieving MRD negativity [110]. The drug was also considered safe, with common toxicities being infections and cytopenias and Grade 1–2 CRS [110]. Larger phase trials are ongoing regarding the role of TEC in earlier lines of therapy, in combination with other agents, and compared to established treatments in the relapse setting. 

If multiple lines of therapy have failed a patient, then multimodal chemotherapy is a reasonable option particularly if the disease is aggressive and immediate control is needed. These regimens often contain agents that a patient has not previously been exposed to in the front-line setting. While this strategy may not induce durable remissions, it will allow for disease control when patients require additional time for the production and manufacturing of a cell therapy product or as a bridge to autologous stem cell transplant. Regimens, or variations thereof, such as DT-PACE or DCEP have been shown to be effective in achieving rapid disease control [111,112]. In heavily pre-treated patients who experience late relapse, there is a role for bendamustine in combination with either bortezomib or lenalidomide based on Phase 1 or 2 data in which approximately 70% of patients achieve a partial response or better [113,114].

Choosing therapy in the Daratumumab refractory setting often implies refractoriness to multiple other agents including PI and IMiDs; clinical trial eligibility should be assessed for all patients in this setting. Decisions to re-treat with DARA, enroll in trials, pursue CAR-T, novel agents such as TEC, versus pursuing chemotherapy-based combinations are often decided based on the aggressiveness of the disease and the availability of a drug in a specific practice setting.

### 3.4. Autologous Stem Cell Transplant

In the relapsed myeloma setting there are two main situations in which there is a role for autologous stem cell transplant (ASCT): in patients who defer ASCT as consolidation in first remission or in patients who have relapsed after front-line ASCT during maintenance therapy. In Table 5, we summarize key clinical trial data supporting the use of delayed or second ASCT for RRMM

Highly effective therapies in the front-line setting, combined with patient preference, frailty, and comorbidities have raised the question in multiple studies of whether ASCT should be conducted in the front-line setting after induction or deferred to the time of first relapse. Data from the Phase 3 DETERMINATION trial showed at a median follow-up of 5 years, superior median PFS (67.5 vs. 46.2 months, HR 1.5, *p* < 0.001) favoring frontline ASCT, but no significant difference in 5-year OS (*p* = 0.99) [115]. Similarly, in the IFM2009 trial, at median follow-up of 93 months, median PFS favored the ASCT group (47.3 vs. 35.0 months, HR 0.70, *p* < 0.001) with median OS not reached in either group, and similar OS rates in the ASCT and no ASCT groups (62.2% vs. 60.2%, HR 1.03, *p* = 0.81) [116]. 

In IFM2009, achieving MRD negativity was a strong predictor of superior outcomes in terms of PFS, PFS2 (progression after next-line therapy), and OS [116]. These MRD-related findings were not seen in DETERMINATION; however, this could be due to a lesser percentage (20–30%) of patients going on to receive ASCT at progression as compared to IFM2009 in which close to 70% of patients received ASCT at progression [115,116]. Ultimately, the findings from both studies support the notion that there is not a one-size fits all approach to ASCT in the first-line vs. second-line setting and that individual patient preference takes priority along with consideration of short- and long-term risks and toxicities related to treatment. 

For patients relapsed after the initial ASCT, the Myeloma X and ReLApsE trials represent the most mature data informing the second ASCT. In the Myeloma X trial, patients who relapsed after ASCT were reinduced with bortezomib, doxorubicin, and dexamethasone and subsequently randomized to ASCT with melphalan conditioning or weekly oral cyclophosphamide [117]. While the re-induction regimen may be considered outdated, patients in the ASCT arm had superior median OS as compared to the cyclophosphamide maintenance group [117]. Similarly, in the more recent German ReLApsE trial, patients received a regimen of LEN-dex reinduction followed by randomization to ASCT with melphalan conditioning followed by LEN maintenance versus LEN-dex indefinitely [118]. In this study, there was a trend toward improved OS; however, it did not reach statistical significance [118]. Study investigators cite that patients harboring high-risk cytogenetic abnormalities were over-represented in the transplant arm as well as about 30% of patients who never received the planned ASCT [118]. Subsequent subgroup analyses of both the Myeloma X and ReLApSE studies confirmed that the overall survival benefit of second ASCT is not seen in subgroups of patients with high-risk cytogenetic abnormalities and is largely limited to standard-risk myeloma patients [119,120]. 

Retrospective real-world data from the Center for International Blood and Marrow Transplant Research (CIBMTR) reported outcomes on 187 patients undergoing second ASCT for relapsed disease between 1995–2008 [121]. Patient outcomes not only support the role of second ASCT in terms of median PFS and OS at 1 and 3 years but also demonstrate safety with non-relapse mortality at 1 and 3 years of 2% and 4%, respectively [121]. Furthermore, this data set was able to identify a specific cohort that might have greater benefit, specifically those that experience relapse >36 months from initial transplant having a longer OS as compared to those relapsed <36 months [121]. More recently, an updated retrospective analysis of CIBMTR data was published by Dhakal and colleagues of 975 patients undergoing second ASCT between 2010 and 2015 [122]. Findings of NRM, PFS, and OS remained consistent as compared to prior analysis [122]. NRM at day 100, 1 year, and 3 years was 1%, 1%, and 2%, respectively [122]. PFS at 1 and 3 years was 50% and 13%, respectively; OS at 1 and 3 years was 94% and 68%, respectively; with significant improvement in PFS/OS in patients relapsing ≥ 3 years as compared to <3 years [122].

### 3.5. Managing Myeloma Relapse with Renal Impairment

Renal impairment is a common feature of multiple myeloma, established as one of the “CRAB” criteria, present in about 50% of patients and associated with higher mortality [123]. Registry data from the United States and Europe have shown that multiple myeloma can contribute to about 2% of cases needing dialysis due to progression to end-stage renal disease [124,125]. Common mechanisms of kidney damage include toxic immunoglobulin light chains causing cast nephropathy, monoclonal protein deposition leading to glomerular damage, and light-chain amyloidosis (AL). In the relapse/refractory setting, whether renal impairment was pre-existing or de-novo, selecting the appropriate next line in treatment can be challenging given the added layer of pharmacokinetic considerations of certain regimens. Furthermore, there is a lack of uniformity amongst clinical trials in RRMM in both inclusions of patients with renal impairment and reporting of renal responses as secondary or exploratory endpoints.

Several active agents in multiple myeloma can be safely dosed in patients with mild to moderate renal impairment. For patients with ESRD requiring dialysis, most medications can also be safely dosed post-dialysis to achieve a therapeutic effect. Out of all of the FDA-approved therapeutics mentioned in this review, generally, most IMIDs, PIs, and anti-CD38 monoclonal antibodies can be dosed safely in patients with renal impairment [126]. Clinical trials referencing subgroups of patients with renal impairment show clinical benefits in terms of OS, PFS, and ORR. The subgroup analyses are not routinely powered to detect differences in terms of efficacy; however, real-world data help support and inform treatment choices [127].

Alongside disease control and progression-free survival, achieving renal recovery with anti-myeloma therapy, measured as improvement in CrCl or eGFR by IMWG criteria, is of equal importance as this correlates with longer OS [128]. The combination of anti-CD38 monoclonal antibody therapy with a PI can be particularly effective. In the ICARIA-MM, in the subgroup of patients with eGFR < 60 (n = 104 patients), complete renal response was achieved in close to twice the percentage of patients in the Isa-Pd arm versus Pd (72% vs. 38%) along with a faster median time to renal improvement [129]. Similar findings were seen in the IKEMA study however with a smaller subgroup of patients with renal impairment (n = 61) [130]. 

While subgroup analyses assessing percent and time to renal recovery with DARA-based regimens have not been conducted from the pivotal Phase 3 CANDOR or CASTOR trials, there is real-world evidence and early phase trial data to support the use of Daratumumab in patients with renal impairment, including dialysis patients [127]. In some cases, patients demonstrated a decrease in dialysis frequency or even liberation from dialysis completely on DARA-based regimens [127,131,132]. The phase 2 DARE trial enrolled RRMM patients with severe renal impairment, defined as eGFR < 30 or on hemodialysis, to a regimen of DARA-dex. In this small cohort of 38 patients, half of the patients were committed to hemodialysis, and of the whole cohort, 17% achieved a renal response by IMWG criteria [133]. At the time of data cutoff, close to 40% of patients were continuing to receive protocol therapy at a median follow-up of 5.5 months; longer-term follow-up is needed [133]. Nevertheless, the design of the DARE study, enriching for patients with renal impairment, gives valuable insight into the effectiveness of anti-myeloma therapy with adequate power and statistical reliability, in contrast to post-hoc subgroup analyses. 

Lastly, a comprehensive evaluation for fitness for autologous stem cell transplant should also be undertaken in the treatment of RRMM. Renal impairment to any degree should not be a contraindication to ASCT as multiple studies have demonstrated that ASCT is safe and effective in patients with renal impairment, even on dialysis. A retrospective review of 475 patients from multiple bone and marrow transplant units in Vienna, Austria between 1998 and 2016 showed no difference in PFS for any stage of renal impairment and no difference in OS for eGFR as low as 45 [134]. A similar retrospective analysis of 370 patients in the UK with all stages of chronic kidney disease undergoing first ASCT found no differences in transplant-related mortality, PFS, or OS as compared to patients with normal renal function [135]. However, worse OS was seen in patients who experienced a decline in GFR of >8.8% at 1 year post-transplant, emphasizing the importance of close monitoring post-transplant in conjunction with a nephrologist [135]. In both the Austrian and UK studies, the extent of renal recovery, as assessed by IMWG criteria, was variable; however, most patients did not experience worsening of renal function for myeloma or non-myeloma reasons 1 year post-transplant [134,135]. The UK study reported a small cohort of eleven dialysis-dependent patients, seven of whom became dialysis-free post-transplant, and four of those seven went on to receive a renal transplant [135]. Multiple studies have demonstrated the reversal of renal impairment to varying degrees in newly diagnosed or relapsed myeloma [136,137].

### 3.6. Managing Myeloma Relapse with Extramedullary Disease

Extramedullary (EM) disease in myeloma occurs on a clinical spectrum that can range from bone-related plasmacytomas, hematogenous seeding of clonal plasma cells in soft tissue causing tumor formation in various organs, or plasma cell leukemia [138]. Clinical presentation can be heterogenous and may be associated with high-risk features and a poor prognosis [139]. In certain situations, patients can have unchanged intact M proteins but markedly increasing serum-free light chains, deemed “light chain escape”. In situations of light chain escape, the toxicity of the light chains can be unpredictable and close attention to vital organ function including kidneys, heart, liver, spleen, pancreas, central nervous system, and skin is imperative. When there is an extramedullary disease at the time of relapse, particularly with plasma cell leukemia, the median overall survival is approximately 6 months or less [139].

Treatment of patients who relapse with EM disease can be challenging as most clinical trials in myeloma exclude these patients, especially plasma cell leukemia. The same principles applied to the management of high-risk relapsed myeloma are applied to managing extramedullary relapse, including consideration of refractoriness to prior lines of therapy. In patients with diffuse visceral disease, rapid cytoreduction is needed and chemotherapy-based regimens such as VDT-PACE, BEAM, or HyperCVAD followed by ASCT or tandem ASCT-allo-SCT have been effective [111,140,141]. Additionally, given that extramedullary disease often harbors high-risk cytogenetic or chromosomal abnormalities, selecting a next-generation PI and IMID such as Carfilzomib and Pomalidomide has been shown to be active [140,142,143]. Data supporting the effectiveness of anti-CD38 monoclonal antibodies such as Daratumumab are limited to case reports and pooled analyses of small subsets of heavily pre-treated patients with EM disease in Phase 1/2 trials, showing improved survival in patients who respond to this strategy [140,144].

Relapse in the CNS, while uncommon, can occur in about 1% of RRMM patients, and the prognosis is extremely poor [138]. Clinical suspicion should be high in any situation where there is a new neurologic deficit with the diagnosis being confirmed via sampling of cerebrospinal fluid or biopsy of a cortical lesion. Treatment approaches should involve the selection of agents that are shown to cross the blood-brain barrier (BBB). High-dose dexamethasone is known to penetrate the CNS, and both lenalidomide and pomalidomide have shown CNS penetration in animal studies; however, no human studies have been reported [138,145,146]. The combination of systemic myeloma-directed therapy, intra-thecal chemotherapy, and CNS irradiation has been shown to improve the duration of response and overall survival [138]. Intra-thecal chemotherapy with either cytarabine or methotrexate in small cohorts resulted in a prolongation of survival by 12–18 months [147,148].

Local radiation therapy or surgical resection should be considered in patients with symptomatic soft tissue or bone plasmacytomas at relapse. Radiation can be effective for local disease control and pain relief depending on the anatomic site. Additionally, surgical resection may be indicated if the plasmacytoma is causing mass effect, organ dysfunction, or axial skeleton or spinal instability. PET/CT imaging is instrumental in determining the extent of extramedullary relapse and monitoring response to therapy.

## 4. Future Directions

The modern era of treatment in RRMM is rapidly evolving, with cellular- and immunotherapy being at the forefront of therapeutic innovation. Treatments such as chimeric antigen receptor T-cell (CAR-T) therapy, bispecific T-cell engagers (BITEs), and Cereblon E3 Ligase Modulators (CELMoDs) are demonstrating encouraging responses in the most heavily of pre-treated patients.

In terms of CAR-T cell therapy, there are currently two FDA-approved CAR-T products, both of which target anti-B cell maturation antigen (BCMA), idecabtagene vicleucel (ide-cel) and ciltacabtagene autoleucel (cilta-cel). For ide-cel, data from the Phase 2 KarMMa study, showed responses in patients having received a median of 5–9 prior lines of therapy, including an IMiD, PI, and anti-CD38 mAb [149]. In the study population, ORR was 73%, with approximately one-third of patients having achieved a CR or better and one-fourth of patients achieving MRD-negativity at 10^−5^ [149]. In terms of survival, at a median follow-up of 13 months, PFS was 8.8 months, and OS not yet reached [149]. Cilta-cel was evaluated in the Phase 1/2 CARTITUDE-1 study in which patients had received a median of six prior lines of therapy [150]. More recent efficacy data at 2 years of follow-up showed an impressive ORR of 98%, with 82.5% of patients achieving a stringent CR within 2–3 months of receiving cilta-cel [151]. With longer follow-up as compared to ide-cel, cilta-cel demonstrated a PFS of 55% with median OS not reached in the overall population [151]. MRD negativity at 10^−5^ was seen in over 90% of patients [151]. On comparison of the two separate CAR-T cell products, the greater magnitude of benefit in cilta-cel over ide-cel could be explained by differences in the study population and post-protocol therapies. It is also important to recognize that despite both constructs targeting BCMA, cilta-cel contains two separate heavy chain domains resulting in enhanced recognition and affinity for BCMA, whereas ide-cel contains only one [151]. Given the impressive benefit of cilta-cel, approaches are currently underway to evaluate it in earlier lines of therapy.

BITE therapy presents a versatile option for RRMM. Preclinical and early phase trials demonstrate efficacy as monotherapy which raises hypotheses of effectiveness in combination with traditional anti-myeloma therapy, as a bridge to transplant, or alternative to CAR-T cell therapy. Currently, there is significant energy towards the study of BITEs, with numerous Phase 1 and 2 trials including novel agents targeting plasma cell surface receptors BCMA, GPRC5D, FcRH5, and CD38, all paired with CD3 recognition to recruit T cells [152]. Out of the flock of available BITE therapies, we highlight Teclistamab, a BITE targeting BCMA and CD3, which recently gained accelerated FDA approval for RRMM patients who have received at least four prior lines of therapy. In the Phase 1–2, MajesTEC-1 trial, the study population receiving Teclistamab had disease refractory to at least two IMIDs, two PIs, and Anti-CD38 mAb, with a median of five prior lines of treatment [153]. At a follow-up of 14 months, ORR was 64%, with close to 40% of patients achieving a CR and sustaining these responses for a median of 18 months [153]. The efficacy of Teclistamab far surpasses the available options in heavily pre-treated patients and presents an attractive option for patients who are not able to access the specialty care or time required for CAR-T cell therapy. 

Lastly, CELMoDs, which are an evolving treatment strategy for myeloma, build upon the long-standing success of IMiDs by using a slightly different molecular structure that allows for more enhanced interaction with traditional IMiD substrates such as cereblon [154]. Two agents currently undergoing clinical assessment include Iberdomide and Mezigdomide. In a Phase 1/2 trial, Iberdomide and dexamethasone had significant clinical activity in heavily pre-treated patients, particularly individuals refractory to both LEN and POM, with ORR of 26–32% [155]. Similarly, Mezigdomide in combination with dexamethasone showed promising activity in an ongoing Phase 1/2 trial with an ORR of 48% at therapeutic doses [156]. While these response rates may not compare in magnitude to response rates seen with BITE or CAR-T, Iberdomide and Mezigdomide represent oral options with favorable safety profiles and foreseeably easier accessibility if proven to be effective in combination with other anti-myeloma therapies. Currently, clinical trials are ongoing for both Iberdomide and Mezigdomide in combination with DARA and BOR with promising clinical activity in recently presented abstracts [157,158].

Despite the promising success of these therapeutics, it is worth noting that in the real-world setting, outside of a clinical trial, access to these therapeutics can be difficult for patients. For instance, with CAR-T cell therapy, logistical issues related to time, CAR-T cell manufacturing, and institutional adaptation, limit its application in a timely manner if at all [159]. Furthermore, access to novel therapies through a clinical trial generally requires establishing care at a large academic medical center, which may not be possible for patients living in rural, underserved, or minority communities. Early partnership with academic medical centers, even prior to relapse, is crucial in promoting access to novel agents, with an effort needed from trial runners and pharmaceutical companies to expand access to these agents to the community and rural practice setting.

## 5. Conclusions

Highly effective therapies for RRMM are helping to control the disease for our patients providing the benefits of improved survival and maintained the quality of life. Relapses continue to occur which is a humbling reminder that the disease remains much smarter than we are as clinicians and researchers. We have managed to outsmart this disease in some ways by leveraging our knowledge of the heterogeneity of plasma cell clones and markers of higher-risk disease and incorporating them into treatment decision-making.

It is well established that next-generation IMIDs such as POM, next-generation PIs such as CAR and IXA, and monoclonals such as DARA, ISA, and ELO will continue to have substantial roles long term in the relapse/refractory setting. Other agents such as VEN and SELI are finding their own niche in very specific situations. Certain pillars of therapy such as ASCT and chemotherapy will continue to exist as options for the right patient in unique scenarios such as renal disease or extramedullary relapse. 

There is promise on the horizon as we race toward a functional cure for myeloma patients, with novel agents such as CAR-T and BITEs showing impressive activity in the most heavy of pre-treated patients, well beyond what was seen two decades ago. As myeloma is viewed more and more as a chronic disease, the key in assessing new therapies will be to answer questions not only related to patients’ survival and response, but also to their quality of life, reported outcomes, financial burdens, and disparities in access to care.

## Figures and Tables

**Table 1:** Relevant IMWG Response Criteria (Adapted with permission from the original publication by Kumar S., et al. [3]).

Response	Definition
Complete response (CR)	Negative immunofixation in the serum or urine and disappearance of any soft tissue plasmacytomas and <5% plasma cells on bone marrow aspirate
Stringent Complete Response (sCR)	Complete response as defined above plus a normal serum free light chain ratio (FLC) and absence of clonal plasma cells on bone marrow aspirate
Very Good Partial Response (VGPR)	Serum and urine M-protein detectable on immunofixation but not on electrophoresis -or- >90% reduction in serum M-protein plus urine M-protein < 100 mg on 24 h collection
Partial Response (PR)	>50% reduction of serum M-protein plus > 90% reduction in or <200 mg per 24 h urine collection If serum and urine M-protein unmeasurable and FLC are also unmeasurable, >50% reduction in plasma cells is required in place of M-protein, provided that the baseline percentage in bone marrow is at least 30%. Additionally, a >50% reduction in the size of soft tissue plasmacytomas is also required as measured by FDG^18^-PET/CT
Minimal Response (MR)	≥25% but ≤49% reduction of serum M-protein and reduction in 24-h urine M-protein by 50–89% If soft tissue plasmacytoma is present, then >50% reduction in size is also required.
Stable Disease (SD)	Not meeting criteria for CR, VGPR, PR, MR, or PD. Generally, not recommended for use as an indicator of response
Progressive Disease (PD)	Increase of 25% from the lowest confirmed response value in one or more of the following criteria: Serum M-protein (absolute increased must be >0.5 g/dL) Serum M-protein increase ≥1 g/dL, if the lowest M component was ≥5 g/dL Urine M-protein (absolute increase must be ≥200 mg/24 h) In patients without measurable serum and urine M-protein levels, the difference between involved and uninvolved FLC levels (absolute increase must be >10 mg/dL) In patients without measurable serum and urine M-protein levels and without measurable involved FLC levels, bone marrow plasma-cell percentage irrespective of baseline status (absolute increase must be ≥10%) ≥50% increase in the size of >1 skeletal or soft tissue lesion, or ≥50% increase in the longest diameter of a previous lesion > 1 cm in short axis (as measured by FDG^18^ PET/CT) ≥50% increase in circulating plasma cells (minimum of 200 cells per μL) if this is the only measure of disease
Clinical Relapse	Any one or more of the following criteria: Direct indicators increasing disease and/or end-organ dysfunction (i.e., CRAB features) Development of new soft tissue plasmacytomas or bone lesions (excluding osteoporotic fractures) Definite increase in the size of existing plasmacytomas or bone lesions by >50% as measured by serial FDG^18^ PET/CT Hypercalcemia (>11 mg/dL) Decrease in hemoglobin of >2 g/dL not related to therapy or other non-myeloma conditions Rise in serum creatinine by 2 mg/dL or more from the start of therapy attributable to myeloma Hyperviscosity related to serum paraprotein
Relapse from CR	Any one or more of the following Reappearance of serum or urine M-protein by immunofixation or electrophoresis Development of >5% plasma cells in the bone marrow Appearance of any other signs of progression (ie. New plasmacytoma or CRAB features)

**Table 2:** Summary of key Phase 3 clinical trials in RRMM in LEN resistant/refractory patient subgroup.

Trial	% LEN Refractory	ORR (%)	PFS	OS
NIMBUS (MM-003) POM-loDex vs. HiDex	92 vs. 95	30 vs. 9	3.9 vs. 1.9 months (HR 0.50, *p* < 0.001)	12.7 vs. 8.0 months (HR 0.73, *p* = 0.0234)
STRATUS (MM-010) POM-loDex	95	32.1	4.6 months	11.9 months
OPTIMISMM POM-BOR-dex vs. BOR-dex	69 vs. 71	82.2 vs. 50.0	11.2 vs. 7.1 months (HR 0.65, *p* < 0.0001)	Data not yet mature
CASTOR DARA-BOR-dex vs. BOR-dex	17.9 vs. 24	80.5 vs. 50.0	9.3 vs. 4.4 months (HR 0.36, *p* = 0.0002)	28.9 vs. 32.6 months (HR 0.96, NS)
CANDOR DARA-Kd vs. Kd	32 vs. 36	90 vs. 67 * and 78 vs. 71 **	28.1 vs. 11.1 months (HR 0.46, *p* < 0.001)	Data not yet mature
IKEMA ISA-Kd vs. Kd	32 vs. 34	NT	NR vs. 15.7 months (HR 0.60, *p* = 0.56)	Data not yet mature
APOLLO DARA-POM-dex vs. POM-dex	79 vs. 80	69 vs. 46 ^¶^	9.9 vs. 6.6 months (HR 0.66, *p* = Sig, NT)	Data not yet mature
ICARIA ISA-POM-dex vs. POM-dex	93.5 vs. 91.5	59.0 vs. 31.4	11.4 vs. 5.59 months (HR 0.593, *p* = NT)	Data not yet mature
ELOQUENT-3 (Phase 2) ELO-POM-dex vs. POM-dex	90 vs. 84	53 vs. 26 ^¶^	10.2 vs. 4.7 months (HR 0.56, *p* = NT) ^¥^	28.3 vs. 16.0 months (HR 0.42, *p* = NT) ^¥^
BOSTON SEL-BOR-dex vs. BOR-dex	37 vs. 39 ^¶¶^	67.5 vs. 53.2	HR 0.63, *p* = NT PFS duration not reported for LEN ref. subgroup	OS duration not reported for LEN ref. subgroup
BELLINI VEN-BOR-dex vs. BOR-dex	20 vs. 28	NT	NR vs. 14.8 months (HR 0.75, *p* = NT	HR 1.82, *p* = NT OS duration not reported for LEN ref. subgroup

Abbreviations: POM—Pomalidomide, loDex—Low dose Dexamethasone, HiDex—High dose Dexamethasone, BOR—Bortezomib, DARA—Daratumumab, ISA—Isatuximib, ELO—Elotuzumab, SEL—Selinexor, VEN—Venetoclax, K—Carfilzomib, NS—Not significant, Sig—Significant, NR—Not Reached, NT—Not Tabulated. * one prior line of therapy, ** two to three prior lines of therapy, ^¶^—Intention to Treat population. ^¥^ Subgroup included dual LEN and PI refractory patients. Individual LEN refractory or PI refractory subgroups not reported. ^¶¶^—Not specified as refractory, only previous LEN exposure.

**Table 3:** Summary of key clinical trials in RRMM with previous BOR exposure subgroup.

Trial	% Previous BOR Exposure	ORR (%)	PFS	OS
ASPIRE * KRd vs. K-dex	62–67 vs. 64–66	NT for subgroup analysis	NT for subgroup analysis	45.9 vs. 33.9 months, HR 0.82, *p* = NT
ENDEAVOR * Kd vs. BOR-dex	54 vs. 54	NT for subgroup analysis	15.6 vs. 8.1 months (HR 0.56, *p* = NT)	41.8 vs. 32.7 months (HR 0.851, *p* = NT)
TOURMALINE MM1 * IRd vs. Rd	69 vs. 69	NT for subgroup analysis	18.4 vs. 13.6 months (HR 0.74, *p* = NT)	53.0 vs. 55.8 months (HR 0.994, *p* = NT)
EMN011/HOVON114 (Phase 2) K-POM-dex	100%	92	26 months	67 months
APOLLO DARA-POM-dex vs. POM-dex	47 vs. 49	69 vs. 46 ^¶^	8.3 vs. 6.3 months (HR = 0.73, *p* = NS, NT)	Data not yet mature
ICARIA ISA-POM-dex vs. POM-dex	76.6 vs. 75.2	60.2 vs. 32.2	11.4 vs. 5.59 months (HR 0.578, *p* = NT)	Data not yet mature
ELOQUENT-3 (Phase 2) ELO-POM-dex vs. POM-dex	78 vs. 82	53 vs. 26 ^¶^	10.2 vs. 4.7 months (HR 0.56, *p* = NT) ^¥^	28.3 vs. 16.0 months (HR 0.42, *p* = NT) ^¥^

Abbreviations: K—Carfilzomib, LEN—Lenalidomide, Dex—Dexamethasone, BOR—Bortezomib, I—Ixazomib, POM—Pomalidomide, DARA—Daratumumab, ISA—Isatuximib, ELO—Elotuzumab, NS—Not significant, Sig—Significant, NR—Not Reached, NT—Not Tabulated. * In ASPIRE, ENDEAVOR, and TOURMALINE MM1, the subgroup defined as previous Bortezomib exposure, not specifically refractory. Subgroups of patients defined as BOR refractory were small (1–2%). ^¶^ Intention to Treat population. ^¥^ Subgroup included dual LEN and PI refractory patients. Individual LEN refractory or PI refractory subgroups not reported.

**Table 4:** Summary of key clinical trials in RRMM with DARA resistant/refractory patient subgroup.

Trial	% DARA Refractory	ORR	PFS	OS
DREAMM-2 Belantamab Mafodotin	100%	32%	2.8 months (95% CI: 1.6–3.6 months)	13.7 months (95% CI: 9.9–NR)
DREAMM-3 BEL vs. POM-dex	NT	41% vs. 36%	11.2 vs. 7 months (HR 1.03)	21.2 vs. 21.2 months (HR 1.13)
MajesTEC-1 Teclistamab	89.7%	63%	11.3 months (95% CI: 8.8–17.1 months)	18.3 months (95% CI: 15.1-NE)*

Abbreviations: DARA—Daratumumab, BEL—Belantamab Mafodotin, POM—Pomalidomide, dex—Dexamethasone, ORR—Overall Response Rate, PFS—Progression Free Survival, OS—Overall Survival, HR—hazard ratio, CI—Confidence Interval, NR—Not Reached, NE—Not estimable, NT—Not Tabulated. * Data not yet mature.

**Table 5:** Summary of key clinical trials in RRMM using delayed or second ASCT.

Trial	ORR	PFS	OS
Myeloma X (ASCT vs. Cy maintenance)	sCR or CR: −39.3 vs. 22.4% VGPR or PR: −43.8 vs. 52.9%	19 vs. 11 months, HR 0.45, *p* < 0.0001	67 vs. 52 months, HR 0.56, *p* = 0.0169
ReLApsE (ASCT + LEN maintenance vs. LEN-dex)	77.9% vs. 74.6% (*p* = 0.57)	20.7 vs. 18.8 months, HR 0.87, *p* = 0.34	NR vs. 62.7 months, HR 0.81, *p* = 0.37

Abbreviations: ASCT—Autologous stem cell transplant, Cy—Cyclophosphamide, dex—Dexamethasone, LEN—Lenalidomide, PFS—Progression Free Survival, OS—Overall Survival, sCR—stringent complete response, CR—complete response, VGPR—very good partial response, PR—partial response, HR—hazard ratio.
